# Advancing automatic text summarization: Unleashing enhanced binary multi-objective grey wolf optimization with mutation

**DOI:** 10.1371/journal.pone.0304057

**Published:** 2024-05-24

**Authors:** Muhammad Ayyaz Sheikh, Maryam Bashir, Mehtab Kiran Sudddle

**Affiliations:** FAST School of Computing, National University of Computer and Emerging Sciences, Lahore, Pakistan; ADAPT Centre, MTU, IRELAND

## Abstract

Automatic Text Summarization (ATS) is gaining popularity as there is a growing demand for a system capable of processing extensive textual content and delivering a concise, yet meaningful, relevant, and useful summary. Manual summarization is both expensive and time-consuming, making it impractical for humans to handle vast amounts of data. Consequently, the need for ATS systems has become evident. These systems encounter challenges such as ensuring comprehensive content coverage, determining the appropriate length of the summary, addressing redundancy, and maintaining coherence in the generated summary. Researchers are actively addressing these challenges by employing Natural Language Processing (NLP) techniques. While traditional methods exist for generating summaries, they often fall short of addressing multiple aspects simultaneously. To overcome this limitation, recent advancements have introduced multi-objective evolutionary algorithms for ATS. This study proposes an enhancement to the performance of ATS through the utilization of an improved version of the Binary Multi-Objective Grey Wolf Optimizer (BMOGWO), incorporating mutation. The performance of this enhanced algorithm is assessed by comparing it with state-of-the-art algorithms using the DUC2002 dataset. Experimental results demonstrate that the proposed algorithm significantly outperforms the compared approaches.

## 1 Introduction

Textual information represents one of the most prevalent forms of communication, with the internet serving as a vast source of textual data. Numerous platforms such as web pages, blogs, micro-blogging sites, user reviews, and forums contribute significantly to the daily production of substantial textual content. Additionally, a wealth of textual information resides in various repositories, including legal documents, scientific papers, biomedical literature, news articles, books, and novels. The accumulation of textual data in both archives and on the internet experiences exponential growth daily.

As of 2019, Google, the predominant search engine, handles a staggering 3.5 billion queries each day, generating hundreds of relevant results pages for every search [1]. Navigating through such an immense volume of data poses a daunting task for information retrieval. Extracting specific information from lengthy documents or multiple sources has become a significant challenge, leading users to invest substantial time in reading and screening materials without the assurance of obtaining the required information [2]. Consequently, text summarization emerges as a crucial solution to distill the essence and central ideas from extensive documents [3]. Manual condensation of lengthy documents is arduous and time-consuming, making ATS the indispensable solution to address this issue [1].

A text summarization system undergoes analysis based on various criteria, which are then classified into three overarching classes: input document, purpose, and output document criteria [4]. Within the input document class, three criteria are further distinguished: input size, specificity, and form. Input size denotes the system’s capacity to process a varying number of documents, ranging from a single document to a group of documents, depending on the application’s nature. Specificity categorizes the document based on whether it pertains to a general or specific domain. The form of an input document may vary concerning structure, genre, scale, and medium.

The second class revolves around the purpose, encompassing factors such as usage, target audience, and the breadth of the anticipated summary. Output document criteria pertain to the generation and bias of the summary.

There are three primary methods of deriving a summary from input units: extractive, abstractive, and hybrid [3]. Extractive text summarization (ETS) involves extracting crucial segments from the source text based on their scores and assembling them to form the target summary. Abstractive Text Summarization, on the other hand, quotes the essence of the documents in the final summary, generating more complex, innovatively written sentences [3]. The hybrid method combines techniques from both extractive and abstractive approaches. Summarization systems can be either neutral or evaluative. An evaluative system incorporates judgments, whether explicitly or implicitly, into the summary. In contrast, a neutral system generates a summary of input units without expressing any judgments [4].

The fundamental architecture of the ATS system comprises three key tasks: preprocessing, processing, and post-processing [1]. During preprocessing, a structured representation of the source units is generated using information retrieval techniques like stemming, stop word removal, segmentation, tokenization, and other similar methods. In the processing phase, various algorithms are employed to convert the input document into the desired summary. Commonly implemented processing strategies include statistical approaches, graph or tree-based methods, machine learning, deep learning, fuzzy logic, ontology, and structure-based techniques. In the post-processing phase, issues such as reordering selected sentences or resolving anaphora are addressed before generating the final summary [1].

An effective summary is a concise, grammatically accurate collection of sentences that encapsulates all crucial sections of the text while maintaining the flow of information and eliminating redundancy [4]. Three key criteria for a good summary are maximum coverage, coherence, and conciseness.

In alignment with the definition of a good summary, our research addresses the following points:

- The produced summary should not surpass the length of the original text.
- Redundancy is to be avoided in summaries derived from one or multiple texts.
- In the case of multi-document input, attention is given to the coherence of the text, ensuring a smooth flow of ideas for the reader.
- The resulting summary must effectively represent the main idea, sparing the reader from the need to peruse the entire document(s).

Historically, researchers have explored various avenues to develop efficient ATS systems. The initial foray involved statistical-based methods, which, while straightforward to comprehend and implement, suffered from limited processing power. Graph-based approaches were subsequently introduced to enhance sentence relevance, yet faced challenges related to language independence and resource-intensive processing. Linguistic approaches yielded improved results compared to statistical methods but were hindered by a lack of suitable natural language processing tools [4].

Addressing the aforementioned limitations, machine learning techniques emerged as a solution due to their capacity to automatically deduce rules. In the discussed techniques, traditional algorithms treated ATS generation as a single objective task. Recognizing the need for a robust and effective algorithm that approaches human-level summary development, our research advocates for considering multiple objectives simultaneously.

Our proposed approach involves an extractive multi-document ATS with the dual objectives of maximizing coverage and minimizing redundancy. To achieve this, we introduce the BMOGWO-M, specifically designed for multi-objective (MO) ATS. Experiments were conducted using the widely utilized DUC 2002 dataset in text summarization tasks. The model’s performance was evaluated using ROUGE-2 and ROUGE-L, with results compared against benchmark methods such as Adaptive DE, NSGA-II, and MOABC.

The significance of this research is that it eliminates the process of navigation through an immense volume of data by extracting specific information from lengthy documents or multiple sources; therefore, ensuring that the users invest their previous time in reading accurate and required materials. The under-considered text summarization system emerges as a crucial solution to distill the essence and central ideas from extensive documents. Manual condensation of lengthy documents is arduous and time-consuming, hence our research serves as the indispensable solution to address this issue. The primary significance of this research is to develop a concise summary that is grammatically accurate and encapsulates all the crucial data while maintaining the right flow of information. In addition to that, our research breaks through the limitation of a single objective machine learning algorithm and designs a MO ATS system. The result implies that the under-considered ATS system outputs a summary with minimum repetition and length but maximum coverage.

### 1.1 Contribution of the research

The research work’s key contributions are outlined as follows:

1. Proposes an extractive multi-document ATS model designed to maximize coverage and minimize redundancy using BMOGWO-M.
2. Proposes BGWO with the integration of a mutation operator to address the multi-document extractive text summarization problem.
3. Conducts experiments on the DUC 2002 dataset, a standard benchmark for text summarization.
4. Evaluate the proposed model’s performance using ROUGE-2 and ROUGE-L metrics.
5. Compares results with benchmark methods, including Adaptive DE, NSGA-II, and MOABC.

### 1.2 Implications of the research

The findings of this research carry numerous implications. Manual summarization is both expensive and time-consuming. By automating the summarization process with advanced algorithms like BMOGWO, organizations can significantly reduce costs associated with human labor while speeding up the process of content analysis and summarization. ATS systems encounter various challenges such as content coverage, summary length determination, redundancy handling, and coherence maintenance. The study’s approach tackles these challenges by incorporating mutation in the BMOGWO algorithm, thereby improving the quality and effectiveness of the generated summaries. The findings of the study have practical implications for industries and domains where the processing of large volumes of text data is essential, such as news aggregation, social media analysis, legal document summarization, and academic research. Implementing enhanced ATS systems can improve decision-making processes and information retrieval tasks in these domains.

The study’s findings can contribute to the development of more refined social profiling techniques [5]. By analyzing text data from multiple sources, including social media posts, news articles, and other online content, researchers can summarize the text and gain deeper insights into individuals’ behaviors, preferences, and attitudes.

The subsequent sections of the paper are organized as follows: In Section 2, an extensive review of pertinent literature is presented, offering insights into the existing body of knowledge on the subject. Section 3 provides a clear exposition of the research problem, defining it and casting it as a MO optimization challenge. The intricacies of the proposed Binary MOGWO with Mutation algorithm are expounded in Section 4, offering a detailed insight into its design and functionality. Section 5 delves into the dataset used for the research, describing the preprocessing steps undertaken and outlining the experimental settings adopted. The findings of the research, along with comparative analyses, are also unveiled in this section, shedding light on the performance and efficacy of the proposed methodology. Section 6 presents limitations of this study and finally, Section 7 encapsulates the research’s concluding remarks, summarizing key insights, implications, and potential avenues for future exploration.

## 2 Literature review

This section is dedicated to exploring ATS and examining the latest techniques deployed in this domain. The challenges of text summarization can be formulated either as single or MO problems. With the continuous evolution of evolutionary computing, MO algorithms are gaining popularity across various fields. A crucial aspect of Multi-Objective Evolutionary Algorithms (MOEAs) involves preserving genetic diversity among individuals to prevent premature convergence.

The proposed MO extractive text summarization technique, as outlined by Sanchez et al. [6], defines multiple objectives. The primary goal is to maximize content coverage, while simultaneously minimizing redundancy, necessitating the optimization of both functions. To achieve this, a MO Artificial Bee Colony (MOABC) algorithm was implemented. Experiments were conducted on Document Understanding Conference (DUC) datasets, and the evaluation metrics employed were Recall-Oriented Understudy for Gisting Evaluation (ROUGE). Sanchez Gomez et al. [7] propose a parallelized approach for the MOABC algorithm, parallelizing its steps and evaluating various parallel programs.

In a comparative study presented in [8], multiple Post Pareto techniques were investigated to identify the optimal solution from the Pareto solutions. Another approach, described in [9], employs MOABC algorithm for multi-document ATS.

Gomez et al. [10] introduced the query-focused crow search algorithm for creating summaries from multi-documents. The experiments indicated a linear correlation between queries and the generated summaries based on sentiment scores. Mallick et al. [11] employ an ensemble summarization technique based on a MOEA utilizing clustering. This approach is used to generate extractive summaries from publicly available biomedical articles, such as those found in MEDLINE.

In addressing the primary challenges of performance and scalability in text summarization systems, Priya et al. [12] propose an Enhanced Text Summarization (ETS) method. To bolster the credibility of ETS, they introduce two forms of MO optimization techniques: discrete and continuous Particle Swarm Optimization (PSO).

Debnath et al. [13] present a method using Cat Swarm Optimization (CSO) for extractive single-document text summarization with a MO focus. The proposed methodology undergoes testing on well-known datasets DUC-2001 and DUC-2002, with evaluation conducted using the ROUGE metric.

In another study by Alami et al. [14], a few ensemble techniques are proposed. The first involves combining Bag of Words (BoW) and word2vec, and the second aggregates information from Bag of Words and neural networks. Autoencoder, Variational Autoencoder, and Extreme Learning Machine are applied, along with voting-based data fusion techniques, on datasets in both Arabic and English.

In their work [16], Mosa et al. introduce a hybrid approach to addressing the challenge of summarizing social media data. They combine PSO with the Gravitational Search Algorithm (GSA) to generate more relevant extractive summaries from a list of messages.

In a broader survey [27], the authors explore various types of text summarization employing Swarm Intelligence techniques. These techniques belong to the family of population-based algorithms inspired by the natural behavior of bees, ant colonies, and flocks of birds. While the survey covers several swarm-based algorithms, the main focus is on Ant Colony Optimization (ACO). The authors also provide a concise survey of recent developments in multi-document and MO research.

To address the extractive ATS challenge, Mojrian et al. propose Multi-document Text Summarization using Quantum-Inspired Genetic Algorithm (MTSQIGA) [19]. In the realm of single-document extractive summaries for the Arabic language, PSO is employed [15], and another approach utilizes a maximum independent set based on the graph [20].

Tomer et al. [21] introduced a firefly algorithm for multi-document text summarization, incorporating a new fitness function. The algorithm was evaluated on DUC-2002, DUC-2003, and DUC-2004 datasets, outperforming other nature-inspired algorithms like PSO and GA based on ROUGE scores. Aote et al. [24] proposed a BPSO and Improved Genetic Algorithm (IGA) for extractive summarization. It used various features and compared favorably with other techniques on the FIRE 2011 Hindi dataset.

Saini et al. introduce a Graph-based features approach for MO summarization [17], evaluating its performance on three datasets. They propose MOOTweetSumm+ as a methodology optimizing various tweet scoring measures together using a binary differential evolution algorithm. In disaster management tweets [18], a MO Binary Differential Evolution (MOBDE) algorithm is applied to optimize objectives simultaneously. The proposed algorithms, MOOTS1, MOOTS2, and MOOTS3, are experimentally shown to demonstrate superior performance, with MOOTS3 outperforming the other approaches.

Verma et al. [22] presented a two-stage hybrid model for text summarization. It employed partitional clustering, normalized Google distance, word mover’s distance, gap statistics, and a teaching-learning-based optimization approach. The proposed method was evaluated on DUC 2001, DUC 2002, and CNN datasets, showing efficacy in generating summaries with improved content coverage. Srivastava et al. [23] presented a summarization approach combining clustering with topic modeling. The method utilized Latent Dirichlet Allocation and K-Medoids clustering, demonstrating superior ROUGE scores on Wikihow, CNN/DailyMail, and DUC2002 datasets. Joshi et al. [25] introduced DeepSumm, leveraging latent information from topic vectors and sequence networks for improved extractive text summarization. The method was evaluated on DUC 2002 and CNN/DailyMail datasets, outperforming existing state-of-the-art approaches in terms of ROUGE-1, ROUGE-2, and ROUGE-L scores. Ghamdi et al. [26] introduced the Graph Convolutional Summarizer, a multi-document summarization method. The proposed method was evaluated on DUC 2004 and DailyMail/CNN datasets, demonstrating comparable performance to state-of-the-art summarization methods based on ROUGE scores.

In conclusion, the literature review underscores the diverse and innovative approaches in text summarization, emphasizing the integration of optimization algorithms and swarm intelligence techniques. Hybrid methods, such as combining PSO and GSA, showcase efforts to enhance the relevance of extractive summaries. The studies on Swarm Intelligence highlight the importance of population-based algorithms, particularly ACO. Proposals like Quantum-Inspired Genetic Algorithms and binary differential evolution address challenges in multi-document and single-document summarization. A summary of related work is presented in Table 1.

**Table 1:** Summary of literature review.

Author, Reference, Year	Algorithm	Dataset	Multi-Objective	Multi-Document
Abdallah et al. [15] 2017	PSO	EASC	No	No
Gomez et. al [6] 2018	MOABC	DUC2002	Yes	Yes
Gomez et. al [7] 2019	Parallelized MOABC	DUC2002	Yes	Yes
Gomez et. al [9] 2020	Decomposition based MOABC	DUC2002	Yes	Yes
Gomez et. al [8] 2019	Post Pareto analysis on MOABC	DUC2002	Yes	Yes
Gomez et. al [10] 2021	Query-focused Adaptive Crow Search Algorithm	TAC2008	Yes	Yes
Mallick et al. [11] 2021	Ensemble learning (NSGA-2, MOHS MOEAD)	PubMed MEDLINE	Yes	No
Priya et al. [12] 2019	Discrete and continuous PSO	Hotel and movie reviews	Yes	No
Debnath et al. [13] 2021	CSO	DUC2001 and DUC 2002	Yes	Yes
Alami et al. [14] 2019	Ensemble learning(Auto encoders and Extreme learning machine) along with data fusion	SKE and EASC	No	Yes
Mosa et al. [16] 2020	Hybrid (PSO and GSA)	25 Arabic Facebook pages	Yes	No
Saini et al. [17] 2020	Differential Evolution	MORESQUE, AMBIENT, and ODP-239	Yes	Yes
Saini et al. [18] 2021	Three version of Binary Differential Evolution	Tweet about Four disastrous events	Yes	No
Mojrian et al [19] 2021	Quantum inspired Genetic Algorithm	DUC 2005 and DUC 2007	Yes	Yes
Uckan et al. [20] 2020	Graph Independent Sets	DUC 2002 and DUC 2004	No	Yes
Tomer et al. [21] 2022	firefly algorithm	DUC-2002, DUC-2003, and DUC-2004	No	Yes
Verma et al. [22] 2022	teaching-learning-based optimization	DUC 2001, DUC 2002, and CNN datasets	No	Yes
Srivastava et al. [23] 2022	Latent Dirichlet Allocation	Wikihow, CNN/DailyMail, and DUC2002	No	No
Aote et al. [24] 2023	Binary PSO	FIRE 2011 Hindi dataset	No	Yes
Joshi et al. [25] 2023	topic vectors and sequence networks	DUC 2002 and CNN/DailyMail	No	No
Ghamdi et al. [26] 2023	sentence embedding and graph structure-aware feature learning	DUC 2004 and DailyMail/CNN	No	Yes

Despite the extensive research conducted in the past, the dynamic nature of textual data and the increasing demand for more efficient and tailored summarization solutions call for continuous innovation. BGWO offers a unique perspective by adapting the well-established GWO algorithm to the binary domain, introducing novel elements to the optimization process. This tailored approach is designed to address the specific intricacies of extractive text summarization, aiming to improve content coverage, reduce redundancy, and enhance overall summarization quality.

## 3 Problem formulation

This section delineates the conceptualization of ATS as a MO optimization problem. Key notations are defined in Table 2, and these notations maintain consistency throughout the paper.

**Table 2:** Notations along with their descriptions.

Notation	Description
*D*	Collection of the documents
$\bar{D}$	Generated summary $\bar{D}\subset D$
*w*	Weight of the corresponding term *t* in *T*
*N*	Total number of documents in *D*
*T*	Set of distinct terms from D *t*_1_, *t*_2_, *t*_3_, ….*t*_*m*_
*s*	Represents a sentence in *D*
*m*	Total number of words in *D*
*n*	Total number of sentences in *D*
*o*_*k*_	Mean vector that represents the main content of *D*

We have a collection of documents *D*, and here we have two approaches to choose from. The first one is to make the collection of documents as a set of sentences *D* = *s*_1_, *s*_2_, *s*_3_, *s*_4_, …..*s*_*n*_ or as a set of documents *D* = *d*_1_, *d*_2_, *d*_3_, *d*_4_, …..*d*_*N*_. This paper selects the first approach and makes the collection in the form of sentences. In our proposed approach, the existence of a sentence *s*_*i*_ is represented by a binary decision variable *x*_*i*_ *ϵ* 0,1. For *x*_*i*_ = 0, the sentence *s*_*i*_ is not a part of the generated summary; otherwise, it is. The representation of the solution is in the form of *X* = (*x*_1_, *x*_2_, *x*_3_, *x*_4_, ……*x*_*n*_).

The generated summary $\bar{D}$ must consider these two goals:

- **Content Coverage**: The generated summary should include core information of the original document, which means that the utmost important and appropriate sentences should be included.
- **Redundancy Reduction**: As we are working on multiple documents for a topic, there could be sentences appearing in different documents. So, the generated summary should avoid including redundant sentences.

### 3.1 Objective 1: Content coverage

The initial objective function, denoted as Φ_*coverage*_(*x*), pertains to content coverage and requires maximization. Content coverage signifies that if a sentence *s*_*i*_ is included in the summary $\bar{D}$, its content coverage reflects the relevance of this sentence concerning other sentences in the document collection. The calculation of Φ_*coverage*_(*x*) is expressed by Eq 1.

$$\begin{array}{c} \Phi_{coverage}\left(x\right)=\sum_{i=1}^{n}sim\left(s_{i},o\right)\cdot x_{i} \end{array}$$ (1)

### 3.2 Objective 2: Redundancy reduction

The second objective function, aimed at reducing repetition, is denoted as Φ_*RedundRed*_(*x*). To capture the concurrent appearance of sentences *s*_*i*_ and *s*_*j*_ in the computer-produced summary, an additional binary decision variable *y*_*ij*_ ∈ 0, 1 is introduced. The value of *y*_*ij*_ is set to 1 if both sentences appear simultaneously in the candidate summary and 0 otherwise. Addressing redundancy involves minimizing the cosine similarity between consecutive sentences *s*_*i*_ and *s*_*j*_. A reduced cosine similarity indicates minimal similarity, signifying that the two sentences are distinct and, therefore, suitable for inclusion in the summary $\bar{D}$. The reduction of cosine similarity is synonymous with the maximization of Eq 2.

$$\begin{array}{c} \Phi_{RedundRed}\left(x\right)=\frac{1}{\left(\sum_{i=1}^{n-1}\sum_{j=i+1}^{n}sim\left(s_{i},s_{j}\right)\cdot y_{ij}\right)\cdot \sum_{i=1}^{n}x_{i}} \end{array}$$ (2)

### 3.3 Vector representation of documents and sentences

Given the efficiency of computer models in handling numerical data, the conversion of textual data into a numerical format becomes imperative for processing. To achieve this, the textual data is transformed into a vector-based representation, and the ensuing techniques employed for this conversion process are expounded upon below.

#### 3.3.1 Mean vector

A mean vector *o*_*k*_ is calculated using the formula below to represent the main content of the document collection *D*.

$$\begin{array}{c} o_{k}=\frac{1}{n}\sum_{i=1}^{n}w_{ik},\;k=1,2,3,4,\ldots m \end{array}$$ (3)

Eq 3 outlines the formula for calculating the mean vector *o*_*k*_ for the document collection *D*. This involves summing up the values for each term across all sentences in *D* and then dividing the result by the total number of sentences, denoted as *n*. Consequently, *o*_*k*_ yields the mean score for each term, expressed as *o*_*k*_ = (*o*_1_, *o*_2_, *o*_3_, …*o*_*m*_).

#### 3.3.2 Cosine similarity

Cosine similarity stands out as one of the most widely employed measures for assessing similarity in textual data, aiding in the identification of similarities between pairs of sentences.

The cosine similarity between two sentences, *s*_*i*_ = (*w*_*i*1_, *w*_*i*2_, *w*_*i*3_, …*w*_*im*_) and *s*_*j*_ = (*w*_*j*1_, *w*_*j*2_, *w*_*j*3_, …*w*_*jm*_), is defined by Eq 4.

$$\begin{array}{c} sim\left(s_{i},s_{j}\right)=\frac{\sum_{k=1}^{m}w_{ik}w_{jk}}{\sqrt{\sum_{k=1}^{m}w_{ik}^{2}\cdot \sum_{k=1}^{m}w_{jk}^{2}}},\;i,j=1,2,3,4,\ldots n \end{array}$$ (4)

#### 3.3.3 Term frequency and inverse document frequency

Consider a sentence *s*_*i*_ = (*w*_*i*1_, *w*_*i*2_, *w*_*i*3_, …*w*_*im*_), where *i* = 1, 2, 3, 4, …, *n*. Each sentence encompasses weights assigned to terms, determined using the *term frequency inverse sentence frequency* denoted as *tf* − *isf*. The *term frequency* signifies the occurrences of a term within a sentence, while the *isf* indicates how many sentences in the document collection contain this term. In essence, *tf* gauges the significance of a term within a sentence, whereas *isf* characterizes the importance of a specific term across the entire document(s). Consequently, *w*_*ik*_ can be computed using the provided formula in Eq 5. This formula is derived from the popular Tf.IDF (Term Frequency. Inverse Document Frequency) weighting scheme used information retrieval.

$$\begin{array}{c} w_{ik}=tf_{ik}\cdot \text{log}\left(\frac{n}{n_{k}}\right) \end{array}$$ (5)

The above two objectives define the goal for the model summary, but the problem is that both goals conflict. Therefore, the MO optimization approach is required to boost content coverage and redundancy reduction simultaneously. We have proposed an optimization algorithm for this purpose which is detailed in the following section.

## 4 Proposed methodology

This section introduces the proposed work of the research, framing Enhanced Text Summarization (ETS) as a MO optimization problem. Our methodology begins with preprocessing the input, encompassing sentence segmentation, tokenization, removal of stop words, and stemming. These preprocessing steps are detailed below.

### 4.1 Input preprocessing

The preprocessing of raw data is a crucial step in the implementation and evaluation of any algorithm. Before applying the MOGWO-M, the raw textual data needs preprocessing to be compatible with the algorithm. The following steps outline the data preprocessing procedures:

1. **Segmentation of Sentences**: The initial step involves segmenting sentences from all documents. This process provides a sentence count for the entire document, facilitating the identification of sentence boundaries.
2. **Tokenization of Words**: Following sentence segmentation, the next step is word tokenization within each sentence. This aids in calculating Term Frequency-Inverse Document Frequency (*tf* − *idf*) scores for the corpus. During tokenization, extraneous characters that do not contribute meaning, such as punctuation, exclamation marks, interrogations, and other non-contributory marks, are removed.
3. **Removal of Stop Words**: Stop words, the most frequently used words (e.g., “a,” “that,” “the,” “it,” “what,” “which,” etc.) that do not add significant meaning to a sentence, are eliminated. A stop words list from the NLTK library, a widely used Natural Language Processing toolkit, is employed for this task. It’s noteworthy that the word “not” is excluded from the stop words list to retain the negation sense in sentences.
4. **Stemming**: After stopping word removal, a Porter stemmer from NLTK is applied to bring each word to its root form. This normalization step ensures that different forms of a word are treated equivalently, enhancing the uniformity of the text data.

**Fig 1:**
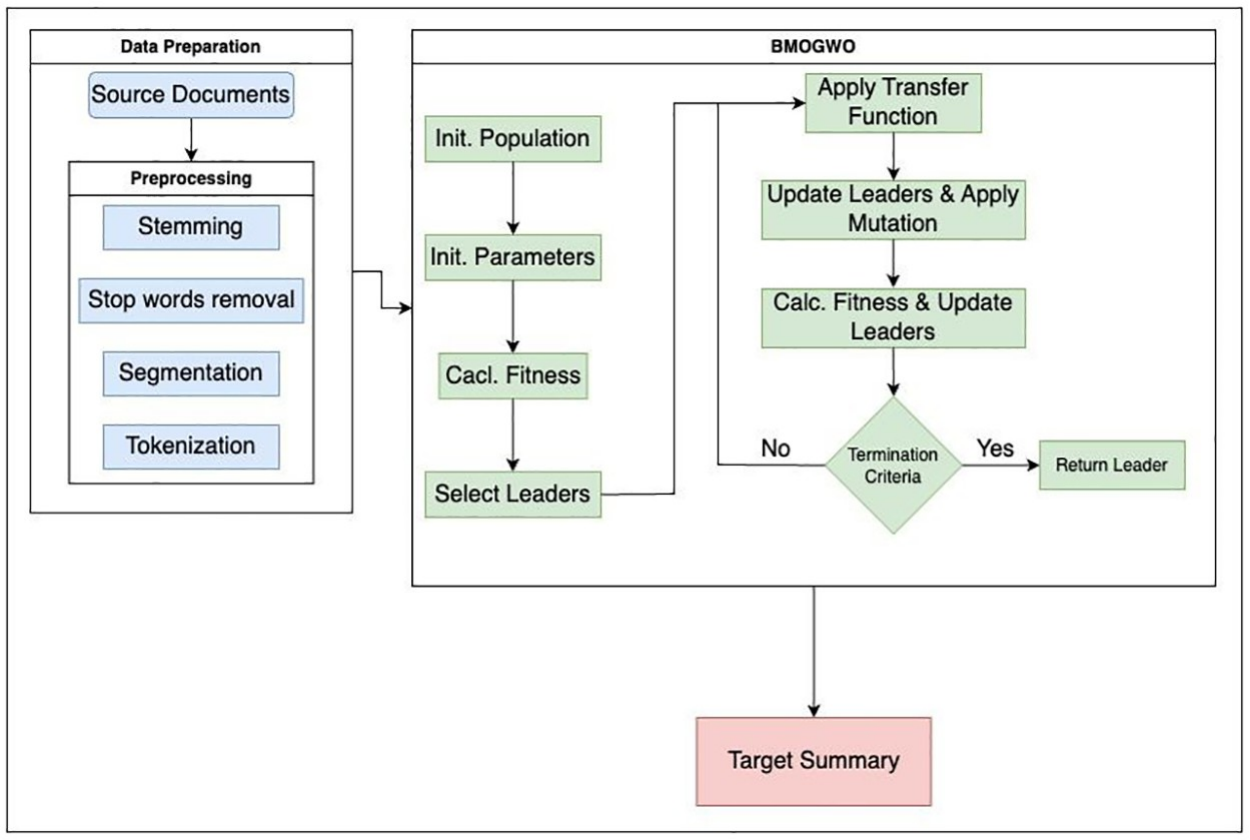
The flowchart of the proposed algorithm.

Following preprocessing, sentences are transformed into vectors, and the document is similarly converted into a vector by averaging the vectors of its sentences. Next, the task of selecting pertinent sentences for summary generation is framed as an optimization problem. To address this, the proposed BMOGWO-M algorithm is employed to solve the optimization problem and choose sentences for summary generation. This process is elucidated in Fig 1.

### 4.2 Binary Multi-Objective Grey Wolf Optimizer with Mutation (BMOGWO-M)

MOGWO, introduced in 2020 [28], represents the latest addition to the class of swarm-based algorithms. Drawing inspiration from nature-inspired techniques observed in grey wolves, MOGWO shares the same working principles as its predecessor, the original GWO. Operating on the hunting mechanism and community-based leadership observed in grey wolves, MOGWO inherits functionalities from the initial GWO model. Originally designed for continuous problems, the MOGWO [29] encountered challenges in direct implementation for discrete problems. To address this, a new module was incorporated, introducing the sigmoid transfer function while keeping the algorithm’s core steps unchanged. We have proposed a binary version of MOGWO with mutation operator. The details of the algorithms are as follows.

In the mathematical representation, the algorithm identifies the three fittest solutions and designates them as *alpha*, *beta*, and *delta*. The most fitting solution is assigned to the *α* wolf, the next fittest to *β*, and the third fittest to the *δ* wolf. The remaining candidate solutions, denoted as *Omega* (*ω*), follow these elite wolves in the optimization iterations to collectively explore and converge towards the global optimum.

Eqs 6 and 7 define the encircling mechanism of wolves during hunting.

$$\begin{array}{c} \vec{D}=\left|\vec{C}\cdot \vec{X_{p}}(t)-\vec{X}(t)\right| \end{array}$$ (6)

$$\begin{array}{c} \vec{X}(t+1)=\vec{X_{p}}(t)-\vec{A}\cdot \vec{D} \end{array}$$ (7)

In the above equations, *t* represents the ongoing cycle, $\vec{X}$ represents the location of the grey wolf in the ongoing cycle, and ${\vec{X}}_{p}$ shows the current location of the prey. The coefficient vectors $\vec{A}$ and $\vec{C}$ are computed with the help of Eqs 8 and 9 respectively:

$$\begin{array}{c} \vec{A}=2\vec{a}\cdot \vec{r_{1}}-\vec{a} \end{array}$$ (8)

$$\begin{array}{c} \vec{C}=2\cdot \vec{r_{2}} \end{array}$$ (9)

The value of $\vec{a}$ reduces linearly within the range of 2 to 0 and $\vec{r_{1}}$ and $\vec{r_{2}}$ are random vectors ranging from [0, 1]. Here, *C* does exploration, and the exploitation initiates if the value of *A* becomes less than 1.

The GWO employs an encircling mechanism and emulates social leadership behavior to explore optimal solutions. The three fittest solutions are designated as leaders (previously defined as *α*, *β*, and *δ*), and the remaining search agents adjust their locations based on Eqs (10)–(16). The parameters *a* and $\vec{A}$ undergo linear changes, influencing the proximity of the *ω* wolves to the prey. When $|\vec{A}|<1$, the wolves converge closer to the prey, while $|\vec{A}|>1$ places them farther away. Ultimately, the score and position of the best solution (*α*) are returned as the outcome of the optimization process.

$$\begin{array}{c} {\vec{D}}_{\alpha }=\left|{\vec{C}}_{1}\cdot {\vec{X}}_{\alpha }-\vec{X}\right| \end{array}$$ (10)

$$\begin{array}{c} {\vec{D}}_{\beta }=\left|{\vec{C}}_{2}\cdot {\vec{X}}_{\beta }-\vec{X}\right| \end{array}$$ (11)

$$\begin{array}{c} {\vec{D}}_{\delta }=\left|{\vec{C}}_{3}\cdot {\vec{X}}_{\delta }-\vec{X}\right| \end{array}$$ (12)

$$\begin{array}{c} {\vec{X}}_{1}={\vec{X}}_{\alpha }-{\vec{A}}_{1}\cdot ({\vec{D}}_{\alpha }) \end{array}$$ (13)

$$\begin{array}{c} {\vec{X}}_{2}={\vec{X}}_{\beta }-{\vec{A}}_{2}\cdot ({\vec{D}}_{\beta }) \end{array}$$ (14)

$$\begin{array}{c} {\vec{X}}_{3}={\vec{X}}_{\delta }-{\vec{A}}_{3}\cdot ({\vec{D}}_{\delta }) \end{array}$$ (15)

$$\begin{array}{c} \vec{X}(t+1)=\frac{{\vec{X}}_{1}+{\vec{X}}_{2}+{\vec{X}}_{3}}{3} \end{array}$$ (16)

To address MO optimization, two key components were incorporated into the GWO. The first component is an archive designed to store non-dominated Pareto solutions generated thus far. The second involves selecting solutions from the archive to serve as leaders (*α*, *β*, and *δ*) for the optimization process. The central element of the archive is the archive controller, which manages entries based on whether the archive is full or a new member seeks inclusion. When a new solution is introduced, it undergoes comparison with existing archive members, leading to four possible cases:

1. If the fitness of the new solution is lower than that of any existing archive member, the new solution is ineligible for archive entry.
2. If the fitness of any archive member is lower than that of the new solution, those member solutions exit the archive, making way for the new solution.
3. If no solution dominates the others, the new solution is allowed entry into the archive.
4. In cases where the archive reaches its capacity, and no space is available for a new solution, a grid mechanism is activated. This mechanism identifies the most occupied area, removes one solution from that region, and allocates space for the new solution. This approach ensures diversity along the Pareto optimal front.

For leader selection, the roulette-wheel method explores the search space to identify less crowded segments. The roles of *α*, *β*, and *δ* are then assigned to non-dominated solutions using this method.

Algorithm 1 provides the pseudocode for the proposed BMOGWO-M. The process begins with the initialization of a population of random grey wolves (*X*_*i*_ where *i* = 1, 2, 3, …, *n*), where binary encoding represents the presence (1) or absence (0) of a sentence in the summary. Each chromosome is represented as *X*_1_ = 0, 1, 1, 1, 0, 0, 1, 0, …, *m*, where *m* denotes the count of sentences in the document collection. Parameters *a*, *A*, and *C* are then initialized. The fitness value for each grey wolf is calculated, and the archive is initiated with non-dominated solutions. The subsequent steps involve leader selection for *α*, *β*, and *δ* wolves, with exclusions to prevent repeated selection. The best solution is assigned to *α*, the second best to *β*, and the third best to *δ*. The last step involves adding *α* and *β* back into the archive.

The iterative phase begins with a while loop that executes until reaching the maximum number of iterations (*Max*_*Iterations*_). Within each iteration, the position of each search agent is updated using Eqs (10)–(16). Subsequently, the positions are binarized using the sigmoid function (Eq 19). Similar operations from the initialization phase, such as updating *a*, *A*, and *C*, are repeated in each iteration. The mutation operation is applied to each search agent before calculating the fitness, contributing to diversified solutions. Two conditional checks are implemented: the first determines whether the archive is full, applying a grid mechanism if necessary, and the second checks if the new solution lies within the hypercube. The leader selection mechanism is then employed for *X*_*α*_, *X*_*β*_, and *X*_*δ*_, and the iteration is incremented.

For the leader selection mechanism, the roulette-wheel method is utilized to calculate probabilities for each hypercube using Eq 17, where *i* represents the segment number, *c* is a constant greater than 1, and *N* is the number of Pareto solutions.

$$\begin{array}{c} P_{i}=\frac{c}{N_{i}} \end{array}$$ (17)

**Algorithm 1**: Binary Multi-Objective Grey Wolf Optimizer with Mutation

Random initialization of the population *X*_*i*_(*i* = 1, 2, 3…*n*)

Assign values to a, A, and C as per their ranges

Find the fitness values of all wolves

Detect non-dominated solutions and initialize the archive

*X*_*α*_ ← SelectLeader(archive)

Remove alpha from the archive

*X*_*β*_ ← SelectLeader(archive)

Remove beta from the archive

*X*_*δ*_ ← SelectLeader(archive)

*t* ← 1;

**while** *t* < *Max*_*iterations*_ **do**

 **while** Each Search Agent **do**

  Update *X*_*i*_ by using Eqs (10)–(16)

  Apply the Transfer function to convert the positions of search agents

  between 0 and 1 by using Eq (19)

 **end**

 Update the values of a, A, and C

 Apply mutation operator by using Eq (18)

 Find the fitness values of all wolves

 Locate non-dominated solutions

 Update the archive as per the new non-dominated solutions

 **if** archive = *archive*_*max*_ **then**

  Invoke the grid procedure

  Add new solution to the archive

 **end**

 **if** *the newly added solution placed outside hypercubes* **then**

  Grid updation is invoked to cover the new solutions

 **end**

 *X*_*α*_ ← SelectLeader(archive)

 Remove alpha from the archive

 *X*_*β*_ ← SelectLeader(archive)

 Remove beta from the archive

 *X*_*δ*_ ← SelectLeader(archive)

 *t* ← 1 + 1;

**end**

return **archive**

### 4.3 Mutation operator

The MOGWO inherently possesses a built-in mechanism for exploring and leveraging the objective space throughout its execution. However, relying solely on exploration and exploitation does not guarantee diversity in the genetic material of a solution. To address this, a mutation operation is introduced to inject diversity, potentially leading to improved solutions. This mutation operation involves adding or removing a sentence from a potential summary based on predefined parameters and criteria.

Let the mutation probability *p*_*m*_ ∈ (0, 1) be a pre-determined value that governs the mutation functionality. For each sentence in the summary, a uniform random number *r*_*i*_ ∼ *U*(0, 1) is generated. If *r*_*i*_ ≤ *p*_*m*_ for a sentence *s*_*i*_ in a summary *x*, then this sentence *s*_*i*_ is considered a valid candidate for mutation. The pseudocode for the mutation operator is presented in Algorithm 2, and the mutation process is defined by Eq 18.

$$\begin{array}{c} sim\left(s_{i},o\right)\geq \frac{1}{n}\sum_{j=1}^{n}sim\left(s_{j},o\right) \end{array}$$ (18)

**Algorithm 2**: Mutation Operator

**function mutate(solution)**

**while** *every sentence in a solution* **do**

 Generate uniform random number r

 **if** *r* ≤ *p*_*m*_ **then**

  **if** $sim\left(s_{i},o\right)\geq \frac{1}{n}\sum_{j=1}^{n}sim\left(s_{j},o\right)$ **then**

   Include this sentence in the solution

  **end**

  **else**

   Exclude this sentence in the solution

  **end**

 **end**

**end**

### 4.4 Sigmoid transfer function

A sigmoid transfer function is introduced to the MOGWO-Mutation to make it workable with discrete problems like ATS. The position update Eq (16) produces continuous values, and to make it compatible with the binary search space, the sigmoid transfer function is used. In the Eq 19, *rand* is a random uniform distribution *ϵ*[1, 0].

$$\begin{array}{c} x_{d}^{k+1}=\{\begin{array}{cc} 1, & If\;sigmoid(\frac{X_{1}+X_{2}+X_{3}}{3})\geq rand.\\ 0, & Otherwise \end{array} \end{array}$$ (19)

## 5 Experiments and results

This section discusses the features of the dataset, experimental settings, and results.

### 5.1 Dataset

To enhance the relevance and comparability of this study with widely used algorithms in ATS, the Document Understanding Conferences dataset (DUC) is selected. DUC is a highly utilized dataset for multi-document text summarization, providing benchmark datasets for evaluation purposes. Specifically, datasets from DUC2002 are employed in this study. The DUC2002 collection comprises various topics, with each topic containing multiple documents extracted from newspapers. Table 3 provides a description and features of DUC2002, while Table 4 offers insights into the topics along with some details.

**Table 3:** Detail of the topics extracted from DUC2002.

Features	Values
Topics extracted from DUC2002	10 (d061j through d070f)
Average documents in each topic	∼8 (from 5 to 14)
Total number of documents	77

**Table 4:** Topic description.

Topic	Number of sentences	Words before preprocessing	Words after preprocessing	Unique words
d061j	183	22598	14610	777
d062j	117	16783	10150	722
d063j	246	28223	17458	957
d064j	183	24388	14933	1023
d065j	289	31940	18595	1196
d066j	201	23229	14192	1027
d067f	122	17236	10880	718
d068f	130	15171	9617	594
d069f	327	48078	30389	1427
d070f	150	19448	12638	704

### 5.2 Experimental settings

This section establishes the parameters, values, and settings for BMOGWO-M, ensuring a fair comparison with MOABC, Adaptive DE, and NSGA-II:

- Number of grey wolves or *pop*_*size*_: 50
- Mutation probability *p*_*m*_: 0.1
- Number of maximum iterations *max*_*iter*_: 1000
- Number of independent runs: 20

The algorithm is executed 20 times to provide a robust evaluation. The experiments are conducted on a MacBook Pro 2020 M1 with 8GB RAM. The implementation is in Python3.8 using Jupyter Notebook. This setup ensures consistency and allows for a comprehensive comparison with other algorithms.

### 5.3 Results

This section discusses details of evaluation metrics, results, and comparisons with recently deployed methodologies.

### 5.4 Evaluation metrics

The summaries are evaluated using ROUGE. DUC has described ROUGE as its official evaluation metric. ROUGE is already prevalent among the tasks related to natural language processing. It measures the model’s performance by comparing the similarity between the human-generated summary and the computed-produced summary. There are different variants of ROUGE, but for this study, ROUGE-N and ROUGE-L are used. For ROUGE-N, bi-gram recall is used to make the comparison by counting the number of overlapping units between the reference summary and the candidate summary. ROUGE-L measures the length ratio of the longest common subsequence between the two summaries.

For a fair comparison with MOABC [6], two more evaluation metrics, range and coefficient of variation, are used. The *Range* is measured using Eq 20 and it gives the information about the dispersion.

$$\begin{array}{c} Range=ROUGE_{best}-ROUGE_{worst} \end{array}$$ (20)

The coefficient of variation (CV) of Pearson is used to define the connection between the range and the mean. The *CV* can be calculated using Eq 21.

$$\begin{array}{c} CV=\frac{Range}{ROUGE_{average}}\cdot 100 \end{array}$$ (21)

### 5.5 Results

The performance of BMOGWO-M is compared with three contemporary MO algorithms: Adaptive Differential Evolution (DE) [30], NSGA-II, and MO Artificial Bee Colony [6]. The evaluation is based on the metrics discussed in the previous section. Tables 5 and 6 present the average, range, and coefficient of variation for *ROUGE* − 2 and *ROUGE* − *L*, respectively, for each topic. This comparative analysis allows for a comprehensive assessment of the effectiveness of BMOGWO-M about other state-of-the-art MO algorithms.

**Table 5:** Average, Range, and CV of ROUGE-2 scores using Adaptive DE, NSGA-II, MOABC, and BMOGWO-M.

Topics	Adaptive DE [30]			NSGA-II [6]			MOABC [6]			BMOGWO-M		
	Average	Range	CV	Average	Range	CV	Average	Range	CV	Average	Range	CV
d061j	0.266	0.290	109.02	0.312	0.260	83.333	0.356	0.090	25.253	0.369	0.046	12.561
d062j	0.188	0.275	146.28	0.220	0.390	177.273	0.346	0.021	6.076	0.342	0.022	6.433
d063j	0.245	0.208	84.90	0.287	0.221	77.003	0.270	0.008	2.963	0.380	0.005	1.343
d064j	0.194	0.280	144.33	0.201	0.397	197.512	0.312	0.010	3.205	0.315	0.007	2.320
d065j	0.144	0.209	145.14	0.185	0.177	95.676	0.211	0.023	10.9	0.269	0.015	5.498
d066j	0.201	0.257	127.86	0.179	0.247	137.989	0.287	0.021	7.317	0.295	0.018	6.145
d067f	0.239	0.235	98.33	0.258	0.287	111.240	0.361	0.006	1.662	0.347	0.021	6.084
d068f	0.491	0.384	78.21	0.499	0.276	55.311	0.427	0.091	21.311	0.478	0.011	2.238
d069f	0.184	0.166	90.22	0.221	0.271	122.624	0.241	0.008	3.320	0.290	0.005	1.831
d070f	0.224	0.260	116.07	0.265	0.213	80.377	0.304	0.003	0.987	0.304	0.002	0.658
Average	0.238	0.256	114.03	0.263	0.274	113.834	0.312	0.028	8.299	0.339	0.015	4.511

**Table 6:** Average, range, and CV of ROUGE-L scores using adaptive DE, NSGA-II, MOABC, and BMOGWO-M.

Topics	Adaptive DE [30]			NSGA-II [6]			MOABC [6]			BMOGWO-M		
	Average	Range	CV	Average	Range	CV	Average	Range	CV	Average	Range	CV
d061j		0.542	0.208	38.380.551	0.201	36.479	0.615	0.051	8.293	0.610	0.029	4.709
d062j	0.473	0.239	50.53	0.492	0.312	63.415	0.534	0.061	11.423	0.653	0.014	2.175
d063j	0.493	0.156	31.64	0.517	0.210	40.619	0.510	0.051	10.0	0.726	0.015	2.012
d064j	0.462	0.235	50.87	0.509	0.213	41.847	0.494	0.013	2.632	0.561	0.011	1.925
d065j	0.431	0.141	32.71	0.511	0.142	27.789	0.468	0.054	11.538	0.578	0.021	3.583
d066j	0.455	0.196	43.08	0.450	0.138	30.667	0.531	0.009	1.695	0.574	0.008	1.341
d067f	0.509	0.232	45.58	0.528	0.215	40.720	0.593	0.015	2.530	0.572	0.038	6.573
d068f	0.666	0.226	33.93	0.624	0.219	35.096	0.641	0.068	10.608	0.697	0.046	6.529
d069f	0.454	0.135	29.74	0.473	0.190	40.169	0.545	0.012	2.202	0.616	0.009	1.461
d070f	0.496	0.173	34.88	0.511	0.155	30.333	0.519	0.007	1.349	0.576	0.006	1.042
**Average**	0.498	0.194	39.13	0.517	0.2	38.713	0.545	0.034	6.227	0.616	0.019	3.135

#### 5.5.1 Comparison of BMOGWO-M with adaptive DE, NSGA-2, and MOABC

Table 5 displays a comprehensive overview of the performance of the proposed BMOGWO-M in comparison to other techniques across 10 different topics. This table presents the average recall, range, and coefficient of variation (CV) values for ROUGE-2, a widely used metric in the evaluation of text summarization systems.

The optimal results are visually highlighted in grey, providing a clear indication of the superior performance achieved by BMOGWO-M in certain scenarios. In particular, BMOGWO-M demonstrates notable improvements in average recall across 7 out of the 10 topics considered. For the remaining three topics, while there are differences, they are marginal. An intriguing observation is seen in Topic d068j, where BMOGWO-M yields a nearly equivalent average recall as NSGA-II. However, it surpasses NSGA-II in terms of range and CV metrics, indicating its potential for better dispersion of results and reduced variability.

Delving into the dispersion metric, CV, it becomes evident that BMOGWO-M exhibits a substantial improvement, achieving a score of 4.51% compared to 8.29%, 113.83%, and 114.03% for the other three techniques. This significant reduction in variation underscores the effectiveness of the proposed methodology in producing consistent and reliable results across multiple runs.

Furthermore, the overall average ROUGE-2 score for all ten topics is higher when using BMOGWO-M compared to the other algorithms considered. This suggests that BMOGWO-M not only excels in individual topic performance but also demonstrates superior overall performance across the dataset.

Analyzing the range metrics for all ten topics, it is notable that BMOGWO-M showcases significant improvement, indicating minimal variance in recall average scores across 20 runs. This consistency further strengthens the credibility of the algorithm’s performance.

**Fig 2:**
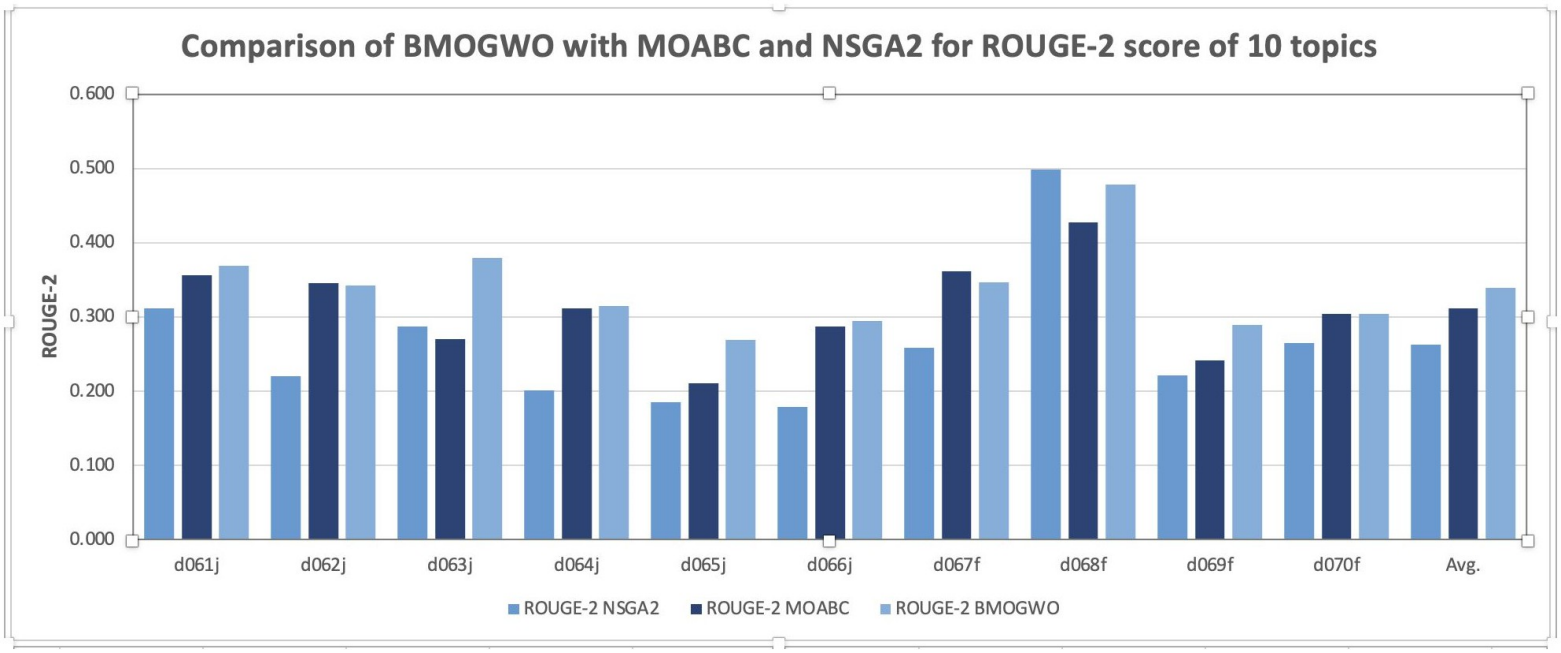
Comparison of BMOGWO-M with MOABC and NSGA2 for ROUGE-2 scores of 10 topics.

To provide a visual representation of these findings, Fig 2 presents a graphical comparison of the average scores of ROUGE-2 evaluation metrics for BMOGWO-M, NSGA-2, and MOABC. Notably, these algorithms stand out from Adaptive DE based on all of the scores, highlighting their effectiveness in comparison. This graphical representation enhances the understanding of the performance differences among the evaluated techniques and reinforces the superiority of BMOGWO-M in achieving higher ROUGE-2 scores and improved dispersion metrics.

Table 6 provides a detailed breakdown of the evaluation metrics for ROUGE-L, focusing on the average recall, range, and coefficient of variation (CV) values across various topics. This analysis sheds light on the performance of the proposed BMOGWO-M methodology in comparison to alternative techniques.

The experimental results showcase notable advancements attributed to the proposed methodology, particularly evident in the enhancement of average recall values across 9 out of 10 topics. Although Topic d067f does not exhibit a substantial improvement, a slight difference of 0.021 in the average recall value hints at nuanced variations in performance across different topic contexts.

Examining the dispersion of results across the 10 topics, the proposed methodology stands out with a CV value of 3.13%. This value significantly surpasses the corresponding CV values obtained by alternative techniques, which are notably higher at 6.22%, 38.71%, and 39.13%. Such findings underscore the consistent and reliable performance of the proposed methodology, suggesting reduced variability in summary quality across multiple runs.

**Fig 3:**
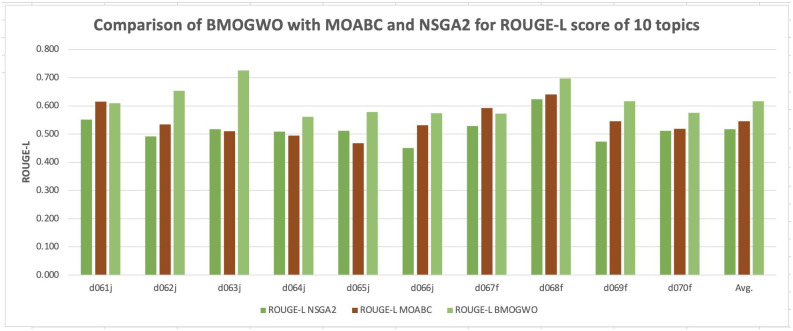
Comparison of BMOGWO-M with MOABC and NSGA2 for ROUGE-L scores of 10 topics.

To enhance comprehension and provide a visual aid for comparative analysis, Figs 2 and 3 offer graphical representations of the comparison between the proposed methodology and other models concerning average ROUGE-2 and ROUGE-L scores, respectively. These visualizations aim to illustrate the performance differentials in a more accessible manner, reinforcing the message that our research yields superior results compared to alternative models. Through these visual depictions, the efficacy of the proposed methodology becomes apparent, further emphasizing its significance in advancing the field of text summarization.

### 5.6 A real world case study

Multi-document extractive text summarization holds great potential in various real-world scenarios. In the media industry, it can be invaluable for extracting pertinent information from multiple documents covering a specific topic. For instance, in the aftermath of a national disaster, where there are numerous digital news documents reporting on the event, extractive text summarization can compile a concise summary of the key points across all articles. Similarly, in the medical field, analyzing the medical records of patients from a particular group can reveal patterns and trends. Extractive text summarization can efficiently summarize these records, aiding in identifying important insights for further study and analysis. In another real-world case study of financial news summarization, our proposed scheme begins with preprocessing raw articles, followed by transforming sentences into numerical vectors and documents into averaged vectors. The process then optimizes sentence selection for summary generation using our BMOGWO-M algorithm. Finally, the summarized news articles are presented to users, aiding them in making informed financial decisions. This demonstrates the scheme’s effectiveness in providing clear and actionable summaries of financial news.

## 6 Limitations of the study

While the proposed BMOGWO-M is rigorously evaluated against state-of-the-art algorithms, the comparison is limited to a specific set of MO optimization techniques. This may overlook potential performance differences with other algorithms not included in the evaluation. The evaluation of BMOGWO-M relies on a specific dataset or datasets, which might not fully represent the diversity of textual content encountered in real-world scenarios. Results could vary with different datasets, potentially affecting the generalizability of the algorithm’s performance. The evaluation primarily focuses on comparing the performance metrics of BMOGWO-M against other algorithms. However, a deeper analysis of factors such as computational complexity, scalability, or robustness could provide additional insights into the algorithm’s suitability for practical deployment.

## 7 Conclusion and future work

Extractive multi-document text summarization poses a challenge that benefits from a MO optimization approach. In the realm of MO optimization, various strategies can be employed to address this task. This study introduces a novel approach, BMOGWO-M, specifically tailored for multi-document text summarization. The formulation of ATS as a MO optimization problem, aiming to maximize content coverage and minimize redundancy, distinguishes this work. Notably, the binary variant of MOGWO with mutation, a unique aspect of this research, has not been explored in the context of multi-document text summarization. BMOGWO-M is implemented and rigorously evaluated against state-of-the-art MO algorithms, namely Adaptive DE, NSGA2, and MOABC. The experimental findings demonstrate the superior performance of the proposed methodology, showcasing its effectiveness in comparison to contemporary algorithms. The continuous exploration and exploitation mechanism inherent in the GWO contribute to its enhanced performance.

In future work, we could explore integrating decomposition techniques into BMOGWO-M, potentially treating the objective functions as a single objective. Additionally, a detailed analysis of the GWO, with a focus on introducing parallelism tailored to ATS, could further enhance its capabilities. Moreover, future research endeavors might include a comprehensive computation time analysis of various algorithms applied to the ATS problem. We could also apply the designed model for creating summaries on real-world datasets such as digital news documents on a national diaster or the medical records of the patients.
