# Correction: Advancing automatic text summarization: Unleashing enhanced binary multi-objective grey wolf optimization with mutation

**DOI:** 10.1371/journal.pone.0305645

**Published:** 2024-06-12

**Authors:** Muhammad Ayyaz Sheikh, Maryam Bashir, Mehtab Kiran Suddle

The third author’s name is spelled incorrectly. The correct name is: Mehtab Kiran Suddle. The correct citation is: Sheikh MA, Bashir M, Suddle MK (2024) Advancing automatic text summarization: Unleashing enhanced binary multi-objective grey wolf optimization with mutation. PLoS ONE 19(5): e0304057. https://doi.org/10.1371/journal.pone.0304057.
